# Neurophilosophical considerations on decision making: Pushing-up the frontiers without disregarding their foundations

**DOI:** 10.3389/fnins.2013.00261

**Published:** 2013-12-30

**Authors:** Gabriel J. C. Mograbi

**Affiliations:** ^1^Department of Philosophy, Federal University of Mato Grosso (UFMT)Cuiabá, Brazil; ^2^Mind, Brain Imaging and Neuroethics Lab, Royal Ottawa Health Care Group, Institute of Mental Health Research, University of OttawaOttawa, ON, Canada; ^3^Coordination for the Improvement of Higher Education Personnel (CAPES)Brasília, Brazil

**Keywords:** decision making, neurophilosophy, moral decisions, testability, ecological relevance, adaptive decision

## Introduction

This is an opinion article on the special research topic now turned into an e-book called “Decision-Making Experiments under a Philosophical Analysis: Human Choice as a Challenge for Neuroscience.” As the first editor of the issue I want to briefly comment on each of the articles highlighting its achievements and prospects for the future.

## The original research section includes 3 articles/chapters

To what extent a decision to deceive someone is conditioned by social pressure of being caught in a lie and suffering the consequences of it? This socially relevant question is addressed in Sip et al. (2012). Deception is a social conduct with practical interests and implications established by complex interaction between interlocutors or agents. Nevertheless, not many empirical studies have been produced so far to understand how the social pressure is internalized by the subjects in their decisions. Sip et al. (2012) explore, in a very creative experimental design, social pressure as a component of decision to deceive. The study makes use of a computer game in which the subject inside the scanner could, in part of the trials, be confronted by an opponent about his/her knowledge of a display's content. A small monetary reward was used to encourage participants to avoid being detected deceiving: Subjects were rewarded for successful deception and penalized for ineffective ventures. The results, in addition to showing, as expected, that the decision to deceive is influenced by the risk of being detected and the social confrontation represented by the detection, also reveal that participants were slower when taking an honest course of action instead of taking advantage of their privileged knowledge. In trials in which confrontation was not possible increased activity in subgenual anterior cingulate cortex was recorded. Also, understanding of a question which allows a deceptive response was associated with activation in right caudate and inferior frontal gyrus.

Deneve (2012) presents an elegant Bayesian decision model that both infers the probability of two different choices and simultaneously estimates the reliability of the sensory information on which this choice is based. Trials in which the level of difficulty is higher show early sensory inputs having a stronger impact on the decision. Accordingly, the threshold collapses such that response time is shorter, tough with lower accuracy. Easy trials, by their turn, show the opposite: an increased sensory weight and a higher threshold over time, eliciting slower, but more accurate, decisions. As the model advanced by the author considers adaptive sensory weights, it could not only extract a single estimate from the sensory input, but also evaluate the uncertainty associated with it. That would be an advantage in comparison to standard diffusion models as it would allow an optimal combination with other noisy sensory cues. The Bayesian model is especially successful when it is possible to encompass prior knowledge with sensory evidence. Notwithstanding its success in monkeys, as human reaction times (RTs) are more asymmetrical than RTs distributions observed in monkeys, traditional diffusion models suit better the human data. Thus, it is still open to further investigation whether the phenomenon is due to the fact that human subjects are less trained than monkeys or because humans may use other cues to evaluate the sensory reliability, not allowing for adaptive sensory gain as, from the beginning, near the optimal value are already achieved.

Osman (2012) empirically compares Choice-based decision making and Prediction-based learning, showing that the former leads to more accurate cue-outcome knowledge. The study mainly focuses on the role of reward. During training period, participants received outcome feedback and were exposed to different types of reward manipulations: Positive Reward, Negative Reward, Both Positive + Negative Reward, No Reward. Negative Reward detrimentally affected Choice-based decision making during learning. By its turn, predictive-based decision making was also negatively affected by Positive Reward. During test period, solely choice was negatively affected by the previously Positive Reward or Negative Reward manipulations exerted in the training period. Based on those results, author suggests that the additional demand of cognitive resources for the processing of rewards could be an explanation of its adverse effect in the decisional process. Also, a series of philosophical considerations is forwarded to question how generalizable is evidence from neuropsychology to psychology and vice-versa. In this context, the relationship of intra-level and inter-level experiments is considered.

In the **Reviews'** section we have a very innovative article by Nakao et al. (2012). This meta-analytical manuscript compares and disentangles two types of empirical protocols used for study of decisional processes: experiments with a unique but uncertain answer and experiments in which no unique external cued answer could be considered correct. The former is categorized as externally oriented decision making and the latter as internally oriented decision-making. The article compares externally and internally guided decision-making empirically and theoretically, studying conceptual and operational differences, as also, similarities between both cases. In the case of externally guided decision, two types of experiments are analyzed: tasks with difficult probabilistic outcome and also experiments in which the answer is varied (or believed to be varied). Both protocols addressing neuroeconomic and social subjects are included in this category. In the case of internally guided decision-making, experiments addressing preference judgment and moral decision making are encompassed. The article uses Multi-Kernel Density Analysis (MKDA) to contrast internally and externally guided decisions in terms of recruitment of areas, to finally compare commonalities and differences between the two types of decisions. The authors show that externally guided decision-making was mainly correlated to the DLPFC-insula-thalamus-IPL network and internally guided decision-making to the VMPFC-pACC-PCC-STG network. Also, it discusses possible future directions to internally guided decision study. Along the contributions to the field of decision making, the article has as one its virtues a contribution to the understanding brain's resting state and its high activity, especially in the Default Mode Network (DMN) that largely overlaps with observed regions in internally guided decision-making.

## In the perspectives section we have 3 chapters

Heinzelmann et al. (2012) discusses the practical and moral question of inappropriate behavior considering its foundations in both philosophical normative and descriptive domains. The moral implication of empirical findings in neuroscience, economics and psychology are discussed in the light of this philosophical background aiming at an understanding of the possible mechanisms of moral inappropriate actions and the decisional process that leads to them. More importantly, the paper addresses the morally important and controversial question of interventions to promote behavior improvement. First, it considers the empirical available knowledge on different techniques of interventions to promote better decisional capacities at various levels of invasiveness: nudging, training, education, pharmacological enhancement and tDCS/TMS. Then, it discusses its feasibility and whether or not we can be morally justifiable to apply those techniques. Both practical and foundational issues are considered to answer this question.

Taking as a standpoint Stephens and Anderson's (2001) already classic article, by Bourgeois-Gironde (2012) aims at considering the viability of methodological transfers from behavioral ecology to experimental economics, including human choice inasmuch as it is concerned with intertemporal preferences. The author suggests that economic theories have noticeable similarities to ecological models in their assumptions and implications. More specifically, it is argued that “hyperbolic time discounting” is present in both humans and other animals, despite the possibility of this process being not only quantitatively but also qualitatively highly different among species. Brief evolutionary considerations are offered to contend for this possibility.

Lucci (2013) proposes an investigation of the subjective component of time in intertemporal choice (IC). The author asserts that deviations from exponential reward discounting, as a function of time, could have as a primary factor the deviation of subjective time from the calendar metric system time. Time perception, she claims, could modulate discounting. Consequently, time perception would be a fundamental component of IC. Reviewing recent literature on time perception, she discusses its relationship with the measuring of IC. Her approach emphasizes the importance of the self in the explanation of behavior from a temporal perspective.

## In the hypotheses and theories axis 3 chapters are presented

Smaldino and Richerson (2012) approached a very important foundational question, namely, the generations of options. The authors argue that current paradigms in neuroscience are focused on decisions made among a previously established set of options, although, the very generation of options has barely been studied and still to a great extent an untapped issue. The author considers various specific factors that could influence the generation of options that would be categorizable in two broadly defined domains: psycho-biological and socio-cultural.

Volz and Gigerenzer (2012) differentiate the “small world” of risk from the “large worlds” of uncertainty. Authors argue that normative strategies used in decisions under risk could not be generalized to all types of decision-making processes, stressing that in most of the experimental designs, the strategies to deal with risk are assumed as implicit presuppositions even if they are not applicable. Also, it is shown that criteria for generating optimal solutions in decisional processes under risk could not be the best whenever uncertainty is the difficulty the agents have to cope with. Even the neural correlates of decision under uncertainty would be different from the ones present in decision under uncertainty. More precisely, value-based statistical thinking would be sufficient for making good decisions in a risk situation but not in the case of uncertainty. Under uncertainty, heuristic thinking would play a key role in an efficient decisional process.

Shadlen and Roskies (2012) argue for the possibility of reconciling responsibility with neurobiology and mechanism by philosophically reviewing presuppositions and implications of recent empirical studies in neurobiology. Instead of the more traditional account of compatibilism based on an appeal to randomness or noise as a source of freedom, they rather recognize that randomness could possibly establish the background against which policies have to be adopted. Although, the argument does not favor compatibility of freedom with determinism *per se*, it contends that compatibilism of responsibility and mechanism is possible. Their arguments function in hypothetical manner: if agents can be accountable for policies that in some sense determine decisions, they can be held responsible for those decisions, even if they do not have conscious access to the reasons for those decisions.
